# Retained Guidewire Following Bedside Femoral Venous Catheterization: A Case Report

**DOI:** 10.1155/cris/4654602

**Published:** 2026-04-09

**Authors:** Yooyoung Chong, Jun Wan Lee

**Affiliations:** ^1^ Department of Thoracic and Cardiovascular Surgery, Chungnam National University Hospital, School of Medicine, Chungnam National University, Daejeon, Republic of Korea, cnu.ac.kr; ^2^ Department of Thoracic and Cardiovascular Surgery, Chungnam National University Hospital, Daejeon, Republic of Korea, cnuh.co.kr

**Keywords:** catheterization, central venous, foreign bodies, fluoroscopy, hemodialysis

## Abstract

Retained guidewire after central venous catheterization (CVC) placement is a rare but preventable complication. Despite advances in ultrasound‐guided catheterization and procedural safety, guidewire retention continues to be reported and may result in vascular injury, thrombosis, embolization, arrhythmia, or infection. We report the case of a 62‐year‐old male who underwent ascending aorta and total arch replacement for acute type I aortic dissection. On postoperative Day 11, a hemodialysis catheter was inserted into the right femoral vein under ultrasound guidance for continuous renal replacement therapy (CRRT). Sequential radiographs obtained within hours demonstrated cranial migration of a retained guidewire from the inferior vena cava (IVC) to the internal jugular vein (IJV); however, the finding was initially misinterpreted as an imaging artifact. The guidewire was recognized ~24 h after insertion and successfully retrieved percutaneously in the angiography suite using a 6‐Fr loop snare under fluoroscopic guidance. Completion venography confirmed intact removal without vascular injury or immediate complication. Although the patient later developed an aorto‐esophageal fistula and died of septic shock on postoperative Day 86, there was no clinical or radiologic evidence linking the retained guidewire or its retrieval to the cause of death. This case highlights the potential for rapid intravascular migration, the risk of radiographic misinterpretation, and the necessity of structured “wire‐out” verification before flushing or securing CVCs, even when ultrasound guidance is used.

## 1. Introduction

Central venous catheterization (CVC) is an essential procedure in critical care, providing vascular access for hemodynamic monitoring, medication administration, and renal replacement therapy. Although generally considered safe, CVC placement is associated with potential complications, including infection, bleeding, pneumothorax, vascular injury, and, rarely, retained guidewire.

The reported incidence of retained guidewire is ~0.03% per catheterization [1]. Despite its low frequency, retained guidewire may result in serious complications such as vascular injury, thrombosis, infection, cardiac arrhythmia, embolization, and migration to major central veins or cardiac chambers. In delayed cases, retained guidewires have required endovascular retrieval or even open surgical removal [2–5].

Because guidewires are thin and flexible, patients may remain asymptomatic for prolonged periods, leading to delayed recognition. Consequently, routine postprocedural imaging review and heightened clinical vigilance are critical to prevent adverse outcomes [6–8].

We present a case of retained guidewire following bedside femoral venous catheterization to emphasize the importance of early detection, careful radiographic review, and preventive strategies during CVC placement.

### 1.1. Case History/Examination

A 62‐year‐old male patient underwent ascending aorta and total arch replacement for acute type I aortic dissection. His medical history included diabetes mellitus, hypertension, and gout. The postoperative course was complicated by acute kidney injury requiring continuous renal replacement therapy (CRRT), as well as hospital‐acquired pneumonia necessitating prolonged mechanical ventilation.

On the eleventh postoperative day (Sunday at 10:00 AM), a malfunctioning hemodialysis catheter in the left common femoral vein was replaced with a new catheter inserted into the right common femoral vein under ultrasonographic guidance. CRRT was resumed without high‐pressure alarms.

The catheter was inserted using the standard Seldinger technique under real‐time ultrasound guidance. After venous puncture of the right common femoral vein, a guidewire was advanced without resistance, followed by serial dilation and placement of a hemodialysis catheter. Adequate blood return was confirmed from both lumens prior to initiation of CRRT. No immediate procedural complications were observed.

A chest radiograph obtained at 13:29 on the same day demonstrated a linear radio‐opaque structure extending from the right common iliac vein into the inferior vena cava (IVC); however, this finding was not recognized at that time (Figure 1A). A subsequent radiograph at 17:27 showed further cranial migration of the guidewire beyond the superior vena cava (SVC), extending toward the right internal jugular vein (IJV). This elongated vertical density was misinterpreted as an imaging artifact (Figure 1B).

Figure 1(A) Postprocedural chest radiograph demonstrating a retained guidewire in the right common iliac vein. (B) Chest radiograph showing migration of the guidewire from the inferior vena cava to the right internal jugular vein.

**Figure:**
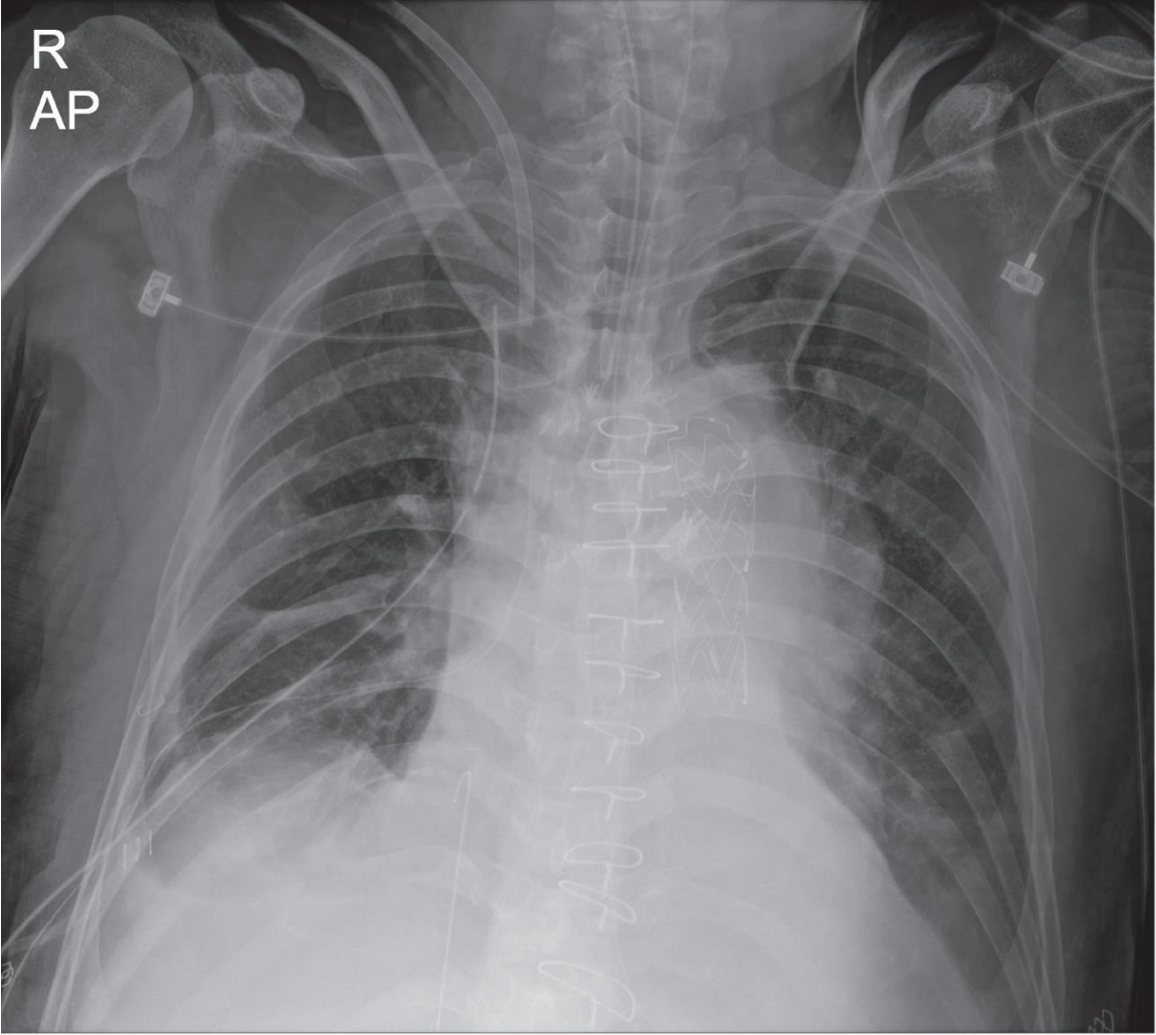
(A)

**Figure:**
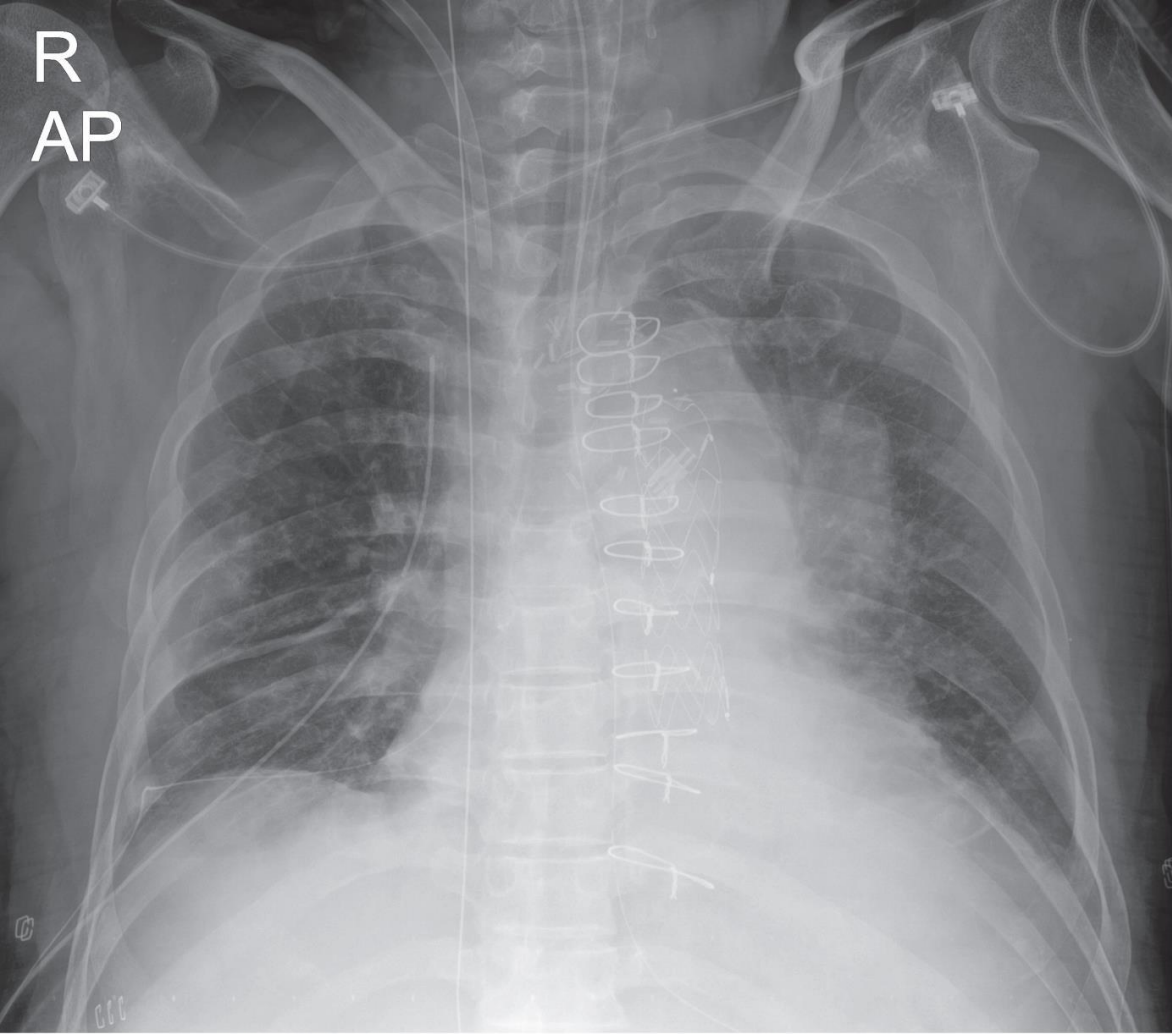
(B)

Thus, migration from the IVC to the IJV occurred within approximately 7 h after catheter insertion. The retained guidewire was ultimately recognized during the Monday morning clinical conference, ~24 h after insertion, when a surgical intensivist identified a linear radiopaque density along the anatomical course of the right IJV, SVC, right atrium (RA), IVC, and right common iliac vein.

Bedside ultrasonography performed immediately after recognition demonstrated a linear hyperechoic structure with posterior acoustic shadowing within the IVC, consistent with an intravascular metallic foreign body. The structure was visualized in longitudinal and transverse planes, confirming the diagnosis of a retained guidewire.

The patient was transferred to the angiography suite for percutaneous retrieval. Under fluoroscopic guidance, a 7‐Fr sheath was inserted into the right femoral vein. Venography confirmed the location of the distal tip of the retained guidewire within the IVC. A 6‐Fr loop snare was advanced through the sheath, and the distal end of the guidewire was successfully captured and withdrawn under continuous fluoroscopic visualization. The guidewire was removed intact without resistance. Completion venography demonstrated no residual foreign body, vascular injury, or contrast extravasation. The patient remained hemodynamically stable throughout the procedure.

Despite this successful retrieval, the patient later developed an aorto‐esophageal fistula and ultimately died of septic shock on postoperative Day 86.

### 1.2. Differential Diagnosis

At the time of detection, differential considerations included radiographic artifacts and retained intravascular foreign bodies. The linear radiopaque density following the anatomical venous course, combined with confirmatory ultrasound findings, established the diagnosis of a retained guidewire.

### 1.3. Conclusion and Results

The retained guidewire was successfully retrieved percutaneously under fluoroscopic guidance without immediate complication. Completion venography confirmed intact removal and absence of vascular injury.

Although the patient later developed an aorto‐esophageal fistula leading to septic shock and death, there was no clinical or radiologic evidence linking the retained guidewire or its retrieval procedure to this outcome. The fistula was considered a known postoperative complication following extensive thoracic aortic surgery.

This case underscores the importance of vigilance, structured procedural verification, and systematic radiographic review to prevent retained guidewires during bedside CVC.

## 2. Discussion

CVC is routinely performed in critical care and is generally considered safe. Nevertheless, retained guidewire remains a rare but preventable complication, with a reported incidence of ~0.03% per insertion [1]. Although many patients remain asymptomatic, retained guidewires may result in serious adverse events, including vascular injury, thrombosis, infection, arrhythmia, and embolization to major central veins or cardiac chambers [3, 4, 6, 9–11].

Because guidewires are thin and flexible, delayed recognition is not uncommon. In the present case, sequential radiographs demonstrated rapid cranial migration from the IVC to the IJV within several hours after insertion. However, the finding was initially misinterpreted as an image artifact, delaying recognition. This observation underscores the importance of careful and systematic radiographic review following CVC placement.

Retained guidewire is widely regarded as a preventable event. Importantly, such incidents are often not due to technical difficulty in obtaining vascular access but rather reflect failure in procedural completion. Prior literature suggests that workflow interruption, distraction, task switching, and omission of a final verification step may contribute to guidewire retention [1, 9]. These mechanisms are typically system‐related rather than solely attributable to individual negligence.

In this case, catheter placement was technically successful under ultrasound guidance, yet guidewire removal was not confirmed at the completion of the procedure. This highlights a critical lesson: successful vascular access does not equate to procedural completion unless guidewire removal is explicitly verified.

To minimize recurrence, a simple but structured “wire‐out” checklist should be performed before flushing or securing the catheter. This may include (1) verbal confirmation that the guidewire has been removed, (2) visual inspection of the removed guidewire to ensure it is intact, and (3) documentation in the procedural note stating, “guidewire removed intact” [12]. Incorporating a brief pause prior to line flushing may serve as a practical safeguard against omission.

Beyond reinforcing established preventive strategies, this case contributes by documenting rapid intravascular migration within hours and by illustrating how retained guidewires may be overlooked when radiographic findings are misinterpreted as artifacts. It also demonstrates that ultrasound‐guided catheter insertion alone does not eliminate the risk of guidewire retention in the absence of structured procedural closure.

This case emphasizes the importance of vigilance, structured procedural verification, and routine imaging review to prevent retained guidewires during bedside CVC.

## 3. Conclusion

Retained guidewire after CVC is a rare but preventable complication. This case highlights that guidewire migration may occur rapidly and can be overlooked when radiographic findings are misinterpreted as artifacts. Strict procedural checks, including explicit confirmation of guidewire removal before catheter flushing or fixation, are essential to prevent this complication.

## Author Contributions

**Yooyoung Chong:** data curation, project administration, resources, writing – original draft, writing – review and editing. **Jun Wan Lee:** conceptualization, data curation, investigation, supervision, writing – original draft, writing – review and editing.

## Funding

No funding was received for this manuscript.

## Disclosure

The authors have nothing to report.

## Consent

Written informed consent was obtained from patients for publication of this case report and any accompanying images.

## Conflicts of Interest

The authors declare no conflicts of interest.

## Data Availability

Data sharing is not applicable to this article, as no datasets were generated or analyzed during the current study.
